# The clinical significance of oestrogen receptor expression in breast ductal carcinoma in situ

**DOI:** 10.1038/s41416-020-1023-3

**Published:** 2020-08-10

**Authors:** Islam M. Miligy, Michael S. Toss, Sho Shiino, Georgette Oni, Binafsha M. Syed, Hazem Khout, Qing Ting Tan, Andrew R. Green, R. Douglas Macmillan, John F. R. Robertson, Emad A. Rakha

**Affiliations:** 1 Division of Cancer and Stem Cells, School of Medicine, University of Nottingham Biodiscovery Institute, The University of Nottingham, Nottingham, UK; 2 Histopathology Department, Faculty of Medicine, Menoufia University, Menoufia, Egypt; 3 The Breast Institute, Nottingham City Hospital, Nottingham University Hospitals NHS Trust, Nottingham, UK; 4 Medical Research Centre, Liaquat University of Medical & Health Sciences, Jamshoro, Pakistan; 5 Division of Graduate Entry Medicine, School of Medicine, University of Nottingham Royal Derby Hospital, Derby, UK

**Keywords:** Breast cancer, Breast cancer

## Abstract

**Background:**

Oestrogen receptor (ER) in invasive breast cancer (BC) predicts response to endocrine therapy (ET) and provides prognostic value. In this study, we investigated the value of ER expression in ductal carcinoma in situ (DCIS) in terms of outcome and the impact on ET decision.

**Methods:**

In total, 643 pure DCIS, diagnosed at Nottingham University Hospitals, were assessed for ER. Clinicopathological data were correlated against ER status, together with assessment of recurrence rate.

**Results:**

ER positivity was observed in 74% (475/643) of cases. ER positivity was associated with clinicopathological variables of good prognosis; however, outcome analysis revealed that ER status was not associated with local recurrence. In the intermediate- and high-grade ER-positive DCIS, 58% (11/19) and 63% (15/24) of the recurrences were invasive, respectively, comprising 7% and 6% of all ER-positive DCIS, respectively. Invasive recurrence in low-grade DCIS was infrequent (2%), and none of these patients died of BC. The ER status of the recurrent invasive tumours matched the primary DCIS ER status (94% in ipsilateral and 90% of contralateral recurrence).

**Conclusion:**

The strong correlation between DCIS and invasive recurrence ER status and the clinical impact of ET justify discussion of the use of ET in ER-positive DCIS treated by breast-conserving surgery. The excellent outcome of low-grade DCIS, which was almost always ER-positive, does not, in the opinion of authors, justify the use of risk-reducing ET. Therefore, the decision on ET for DCIS should be personalised and consider grade, ER status and other characteristics.

## Background

In countries with routine mammographic screening, one case of pure ductal carcinoma in situ (DCIS) is diagnosed for every four cases of breast cancer (BC).^1^ The management of DCIS continues to be a challenge. Although DCIS is predominantly associated with a low risk of mortality,^2,3^ it is well documented that it could progress into invasive BC (IBC), with an associated increased mortality risk.^4,5^ Precise identification of the mortality risk has been difficult as most of the studies do not provide long-term (i.e. >20 years) mortality data. For example, a woman in her 40s or 50s treated with breast-conserving surgery (BCS) who subsequently died following DCIS would normally develop an initial local invasive recurrence, then subsequently metastatic disease before eventually dying of BC. Furthermore, in the last 20 years, the average survival of patients with ER-positive primary or metastatic BC has increased. In addition, the psychological impact of a recurrence, in situ or invasive disease, must also not be underestimated. Currently, lack of a robust tool to identify low-risk DCIS results in recommendation that all women with DCIS undergo treatment. The results of clinical trials that provide no active treatment to low-risk DCIS are awaited.^6,7^

The optimal clinical management for women with newly diagnosed DCIS is controversial, with variable patterns of practice.^8–10^ The standard management options for the treatment of DCIS in the United Kingdom currently are mastectomy or BCS with or without post-operative whole-breast radiotherapy (RT).^11^

Approximately 70% of women with DCIS will be treated with BCS followed by RT, because of its proven efficacy to reduce local recurrence (LR) risk.^12–14^ RT may be omitted for women at low risk of recurrence; however, clinical and pathological features have not reliably identified patients at low risk of LR following BCS alone, leading to variability in treatment and outcomes of women with DCIS.^15^

Data on adjuvant endocrine therapy (ET) in DCIS continue to evolve debate. Its use has become more common in the United States, with approximately 60% of all DCIS cases having BCS receiving it in recent years.^16^ Adjuvant ET for hormone receptor-positive DCIS tumours, with tamoxifen or aromatase inhibitors (e.g. Anastrozole), may improve local control in hormone-responsive disease^12,17–20^ and reduce the risk of BC recurrence, but survival benefit is unproven.^4,12^ Data on outcome by oestrogen receptor (ER) status of the DCIS are absent in most randomised trials. However, a sub-study of 732/1,799 (41%) of patients in the NSABP B-24 trial reported that tamoxifen significantly decreased BC recurrence in ER-positive but not ER-negative DCIS.^21^

The National Institute for Health and Care Excellence (NICE) has updated the recommendations for adjuvant ET for DCIS to offer it after BCS for women with ER-positive DCIS if RT is recommended but not received, and to consider ET after BCS for women with ER-positive DCIS if RT is not recommended.^22^ The American Society of Clinical Oncology/College of American Pathologists also recommend testing of DCIS for ER to determine the potential benefit of ET to reduce risk of future BC.^23^ Although the concept of ET for ER-positive DCIS patients who require RT, but cannot or choose not to receive it, is obvious as these patients are usually at high risk of disease progression and/or recurrence, this is currently a relatively small group. In reality, most women suitable to receive ET for DCIS also receive RT and/or are at low risk.^16^ Moreover, clinical application of these recommendations means that ER testing would be necessary for all DCIS in order to be considered for management decision. However, DCIS is not routinely stained with ER, and the impact of ET on the outcome of DCIS, and whether this impact is limited to ER-positive DCIS, remains to be defined. Furthermore, routine measurement of ER would increase the burden on the pathology service. The effect of implementation of such recommendation on clinical practice, and the effect on overall patient mortality and morbidity, should be investigated.

In this study, we used a large retrospective cohort of DCIS treated in a single institution to address the outcome of ER-positive DCIS, especially invasive recurrence to consider the utility of routine ER testing in these patients. We have also reviewed the randomised studies reporting on adjuvant ET and clinical outcomes of DCIS.

## Methods

A pure DCIS cohort (*n* = 1249) diagnosed at the Nottingham University Hospitals NHS Trust over a 30-year period (1990–2017) with at least 5 years of follow-up time was identified. DCIS associated with invasive or microinvasive carcinomas was excluded. All demographic, clinical, pathological and outcome data were retrieved from patients’ records. Nuclear grade was assessed using the previously published criteria.^24–26^ In this study, glass slides from all cases were reviewed histologically by an observer and graded according to World Health Organisation (WHO) criteria of breast tumours classification. Grade was compared with the originally reported grade, and conflicted cases were reviewed by a consultant pathologist. Cases with more than one grade were reported, and the higher grade was considered in the final analysis. Management details, including the operation type (BCS or mastectomy), and RT data, were collected. Over the period of the study, the management of DCIS showed significant changes with an increase in the rate of BCS over mastectomy, and more frequent use of local RT as previously described.^27^ ER status was not routinely assessed in DCIS, unless indicated for diagnostic purposes, and was not used to guide further management. Ten- and 15-year ipsilateral BC tumour recurrence (BCTR) was defined as any event of ipsilateral local tumour recurrence (either as DCIS (DCIS–BCTR) or invasive disease (I-BCTR)) occurring after 6 months from the first DCIS surgery and up to 120 and 180 months, respectively. Contralateral BC (CBC) was assessed and defined as any contralateral breast event, either DCIS or IBC, identified after the primary diagnosis of DCIS. BC- specific survival was defined as the time from the primary diagnosis of DCIS to death from BC. Patients were censored at the last time they were seen alive, died of other causes or the time they were lost to follow-up. ER status of the invasive recurrences, either ipsilateral or contralateral, was available as part of the routine management of patients.

In this study, ER status was assessed retrospectively on tissue microarray (TMA) sections of DCIS sample using immunohistochemistry for research purposes, as previously described.^28^ Briefly, 4-µm sections were stained on the diagnostically valid Ventana Benchmark^®^ ULTRA system (Tucson, Arizona, USA) using Ventana anti-ER (SP1) rabbit monoclonal primary antibody as per the recommended protocol. Sections were deparaffinised, and antigen retrieval was performed with a cell conditioner 1 (CC1) for 60 min. The primary antibody was applied for 16 min at 37 °C followed by the OptiView HQ Linker for 8 min and the OptiView HRP Multimer for 8 min. Counterstaining was performed with Mayer’s haematoxylin. Positive control cores were added to each TMA section. Only nuclear staining of DCIS cells was scored. ER positivity was considered when ≥1% of DCIS cells showed nuclear staining.^23^ The final number of cases that were suitable for assessment for ER was 643/1249 (51%) cases, according to the availability of tumour tissue and informative cores in the TMA blocks. Data on progesterone receptor (PR) and HER2 status based on retrospective staining of the TMA cores are available as previously described.^27–29^

### Statistical analysis

Statistical analyses were performed using SPSS v26 (Chicago, IL, USA) for Windows. The association between ER status and clinicopathological parameters was evaluated using Chi-squared test. ER-associated risk with ipsilateral and contralateral recurrence was evaluated individually, as well as the overall risk combining both ipsilateral and contralateral events. Univariate survival rates were determined using the Kaplan–Meier method and compared by the log-rank test. A multivariate Cox regression hazard model was used to adjust confounding factors. All tests were two-tailed, and a *P* value of <0.05 was considered as statistically significant.

## Results

### Study cohort

**Fig. 1:**
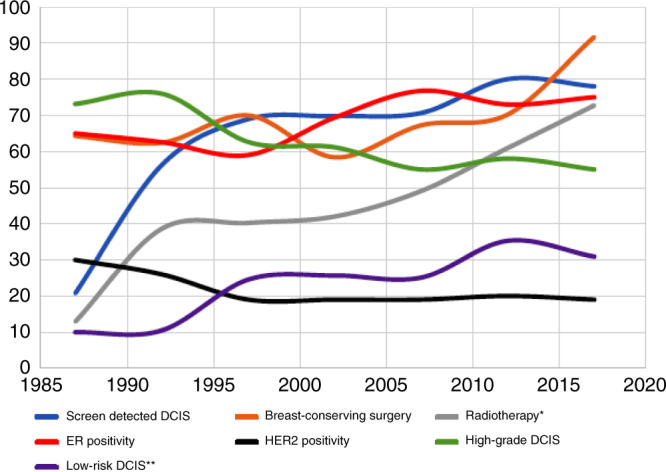
The annual rates of the various clinicopathological parameters of the study cohort, over the period between 1987 and 2017. The graph shows a slight increase in oestrogen receptor (ER) positivity rate over the time accompanied by a quite similar change in breast-conserving surgery (BCS) rates. There was a steady increase in screen-detected DCIS and radiotherapy rates over time. There were slightly lower rates of high-grade DCIS from the start of the study till the end, which were reflected on HER2 positivity rate. Low-risk DCIS rate increased over time as well. *Radiotherapy rate for BCS-treated patients only, **DCIS risk estimated based on tumour size, grade and age at diagnosis.

A total of 475/643 (74%) DCIS cases showed positive expression of ER defined as ≥1% of tumour cells showing nuclear positivity. In this study, only four cases (0.8%) showed ER positivity in 1–10% of tumour cells. There was a trend towards an increase in ER positivity over the period of the study (Fig. 1). This was accompanied by an increase in the rate of screen-detected DCIS and lower rate of high-grade DCIS (Fig. 1). High nuclear grade was observed in 60% of cases (388/643), while comedo necrosis was present in approximately two-thirds of cases (64%). In total, 300 (47%) patients were treated by BCS, while one-third of them received RT (100/300). Over the period of the study, there was an increase in the rate of BCS as a primary surgical choice and rate of offering RT (Fig. 1).

### Association between ER and other clinicopathological factors

ER positivity was associated with features of good prognosis, including smaller tumour size (<40 mm), low nuclear grade, absence of comedo necrosis, positive PR status and lack of HER2 overexpression (all *P* < 0.0001). ER-positive DCIS patients were more likely to be treated with BCS (*P* < 0.0001), without adjuvant RT (*P* = 0.039) compared with ER-negative cases (*P* < 0.0001). Table 1 summarises the correlations between ER expression and other clinicopathological parameters.

**Table 1:** Correlation between ER expression and the clinicopathological variables of DCIS cases.

Parameter	ER expression			
	Total (*n* = 643) *n* (%)	Negative (*n* = 168) *n* (%)	Positive (*n* = 475) *n* (%)	*χ*^2^ (*P* value)
*Age (years)*^a^				
<40	23 (4)	6 (26)	17 (74)	0.077 (0.962)
40–60	354 (55)	94 (27)	260 (73)	
>60	266 (41)	68 (26)	198 (74)	
*Presentation*				
Screening	336 (52)	96 (29)	240 (71)	2.178 (0.140)
Symptomatic	307 (48)	72 (23)	235 (77)	
*Size*^a^				
<16 mm	210 (33)	42 (20)	168 (80)	17.272 (<**0.0001**)
16–40 mm	248 (39)	57 (23)	191 (77)	
>40 mm	182 (28)	68 (37)	114 (63)	
*Grade*				
Low	88 (14)	6 (7)	82 (93)	25.194 (**<0.0001)**
Intermediate	165 (26)	10 (6)	155 (94)	
High	390 (60)	152 (39)	238 (61)	
*Comedo necrosis*				
Yes	412 (64)	145 (35)	267 (65)	48.844 (**<0.0001**)
No	231 (36)	23 (10)	208 (90)	
*Management*^b^				
Mastectomy	342 (53)	110 (32)	232 (68)	13.617 (**<0.0001**)
BCS	300 (47)	58 (19)	242 (81)	
*Radiotherapy*^c^				
Yes	100 (33)	26 (26)	74 (74)	4.275 (**0.039)**
No	200 (67)	32 (16)	168 (84)	
*PR status*				
Positive	342 (58)	4 (1)	338 (99)	274.791 (**<0.0001)**
Negative	246 (42)	154 (63)	92 (37)	
*HER2 status*^d^				
Negative	447 (81)	73 (16)	374 (84)	84.379 (**<0.0001)**
Positive	107 (19)	63 (59)	44 (41)	

*DCIS* ductal carcinoma in situ, *N* number, *X*^2^ Chi square, *ER* oestrogen receptor, *BCS* breast- conserving surgery, *PR* progesterone receptor.

^a^Age and size: categorised according to the Van Nuys Prognostic Index (VNPI).

^b^Management is according to the final operation.

^c^Radiotherapy status is for cases treated with BCS.

^d^HER2 final status is achieved using a combination of IHC and chromogenic in situ hybridisation (CISH).

*P* values in bold are significant.

### Ipsilateral local recurrence and ER status

**Fig. 2:**
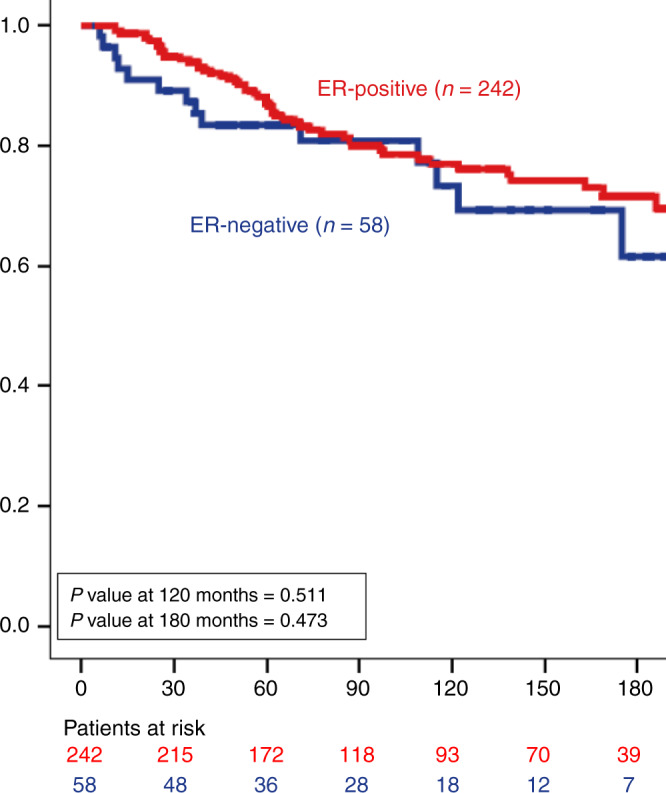
Kaplan–Meier curve shows the association between oestrogen receptor (ER) expression and ipsilateral local recurrence rate in patients treated with breast-conservative surgery (horizontal axis: local recurrence- free interval in months, vertical axis: probability of recurrence). Number of cases at risk after 15 years becomes smaller for meaningful statistical analysis.

The number of cases who developed ipsilateral local recurrence (ILR) over a period of 10-year follow-up was 61 (9%), of which 35 patients (57% of recurred cases and 5% of the overall cohort) developed invasive ILR. In all, 56 cases (92% of all recurrences) recurred after BCS (with or without RT) (56/300, 19%) and only 5 cases (8% of all recurrences) occurred after mastectomy (5/343, 1%). Within the BCS-treated group, 55% of recurrences (31/56) were invasive recurrence. No statistically significant difference was observed between ER status and ILR in patients treated with BCS at 10-year (*P* = 0.511) and at 15-year follow-up (*P* = 0.473) (Fig. 2). Similar results were shown when the analysis was carried out on the whole cohort, regardless of the surgical management (Supplementary Fig. 1), and in multivariate analysis with other confounder factors, including age at diagnosis, tumour size, grade and RT (Supplementary Table 1). In ER-positive cohort, recurrence was mainly associated with nuclear grade and RT (Table 2). RT improved the outcome in the whole cohort and in ER-positive DCIS (*P* = 0.039 and *P* = 0.040, respectively).

**Table 2:** Correlation between various clinicopathological factors and recurrence in ER-positive DCIS treated with breast-conserving surgery.

Parameter	ER-positive DCIS in BCS-treated patients (*n* = 242)		
	No recurrence (*n* = 190)	Recurrence (*n* = 52)	*χ*^2^ (*P* value)
*Age (years)*^a^			
<40	3 (2)	3 (6)	2.979 (0.226)
40–60	101 (53)	26 (50)	
>60	86 (45)	23 (44)	
*Presentation*			
Screening	121 (64)	26 (50)	3.206 (0.073)
Symptomatic	69 (36)	26 (50)	
*Size*^a^			
<16 mm	101 (53)	28 (54)	0.127 (0.966)
16–40 mm	76 (40)	20 (39)	
>40 mm	13 (7)	4 (7)	
*Grade*			
Low	46 (24)	4 (8)	7.028 (0.030)
Intermediate	61 (33)	19 (36)	
High	82 (43)	29 (56)	
*Comedo necrosis*			
Yes	104 (55)	24 (46)	1.207 (0.272)
No	86 (45)	28 (54)	
*Radiotherapy*			
Yes	68 (36)	6 (12)	11.311 (0.001)
No	122 (64)	46 (88)	
*Margin status (mm)*			
<2	9 (5)	2 (4)	0.058 (0.971)
≥2	169 (95)	44 (96)	
*PR status*			
Positive	144 (83)	38 (84)	0.038 (0.846)
Negative	29 (17)	7 (16)	
*HER2 status*^b^			
Negative	125 (85)	40 (84)	0.011 (0.915)
Positive	23 (15)	7 (16)	

*DCIS* ductal carcinoma in situ, *N* number, *χ*^2^ Chi square, *PR* progesterone receptor, *ER* oestrogen receptor.

^a^Age and size: categorised according to the Van Nuys Prognostic Index (VNPI).

^b^HER2 final status is achieved using a combination of IHC and chromogenic in situ hybridisation (CISH).

*P* values in bold are significant.

In all, 80% of patients who developed invasive ILR within 10 years, were initially treated for ER-positive DCIS (28/35). Data on the ER status of the invasive ILR (*n* = 30) showed that 94% of these tumours had the same ER status as the primary DCIS. Twenty-two out of 23 ER-positive DCIS patients developed ER-positive invasive ILR (96%), whereas six out of seven ER-negative DCIS patients developed ER-negative invasive carcinoma (86%). The discrepant ER-positive case was a patient who had intermediate- and high-grade DCIS, who subsequently developed ER-negative invasive disease, which was grade 3 ductal carcinoma of no special type (NST) associated with high-grade DCIS (which was most likely a new primary). The discrepant ER-negative case was high-grade DCIS with a triple-negative phenotype, whereas the subsequent tumour was ER-positive invasive lobular carcinoma, which could be representative of a new primary rather than being a true recurrence from the primary DCIS tumour.

### Outcome of ER-positive DCIS based on nuclear grade

ER positivity was more frequent in low- and intermediate-grade DCIS than high grade. Forty-four patients who had ER-positive DCIS and were treated with BCS developed ILR within 10 years. In all, 59% (26/44) of these were invasive disease (11 cases followed intermediate-grade DCIS and 13 cases followed high-grade DCIS). Two low-grade ER-positive DCIS recurred as invasive disease (2% of low-grade DCIS), and both recurrences were low-grade ER-positive invasive carcinoma associated with low-grade DCIS. Both patients were alive at the end of follow-up; the 10-year survival rate in this group was 100%. Table 3 summarises the percentage of ER-positive cases within the different grades of the DCIS cohort.

**Table 3:** Oestrogen receptor status among the different DCIS grades and the corresponding recurrence rate.

DCIS grade	Number of ER-positive cases (*n* = 475, 74%)	Ten-year overall (DCIS and invasive) recurrence rate in the ER-positive group (*n* = 47, 10%)	Ten-year invasive recurrence rate (*n* = 28, 6%)	ER-positive invasive recurrence (*n* = 22, 5%)	Overall recurrence (DCIS and invasive) rate in the ER-positive group (*n* = 55, 12%)	Overall invasive recurrence rate (*n* = 34, 7%)	ER-positive invasive recurrence (*n* = 25, 5%)
Low (*n* = 88, 14%)	82 (94% of low grade)	Four cases (5% of all low-grade ER-positive cases)	2 (2%)	2 (100%)	Five cases (6% of all low-grade ER-positive cases)	3 (3%)	Two cases with available ER data were all ER-positive (100%)
Intermediate (*n* = 165, 26%)	155 (94% of intermediate grade)	19 (12% of all intermediate-grade ER-positive cases)	11 (7%)	Nine cases with available ER data were all ER-positive (100%)	20 (13% of all intermediate-grade ER- positive cases)	11 (7%)	Nine cases with available ER data were all ER-positive (100%)
High (*n* = 390, 60%)	238 (61% of high grade)	24 (10% of all high-grade ER-positive cases)	15 (6%)	11/12 cases with available ER data were ER-positive (92%)	30 (13% of all high-grade ER-positive cases)	20 (8%)	14/15 cases with available ER data were ER-positive (93%)

The invasive recurrence rates in the ER-positive intermediate- and high-grade DCIS groups were 7% and 6%, respectively. The ER status of the invasive recurrence was 100% identical for the intermediate-grade group, while 92% of invasive recurrences that occurred after primary diagnosis of high-grade ER-positive DCIS showed similar ER positivity.

Interestingly, the figures of the overall recurrence rate up to the end of the follow-up period for each nuclear grade were comparable with the 10-year recurrence rate in terms of ER-positive recurrences (Table 3).

### Contralateral breast cancer and ER status

Contralateral BC (CBC) was identified in 55/643 cases (9%), of which 37 cases were invasive disease (representing 6% of the overall cases and 67% of the contralateral events). In patients initially treated for ER-positive DCIS, 30 cases had invasive CBC. The primary DCIS in those cases were high grade in 14 patients (47%), intermediate grade in 11 (34%) and low grade in 5 cases (17%). In all, 90% (27/30) of the contralateral invasive disease cases were ER-positive. The three discrepant cases that developed ER-negative contralateral invasive disease (high-grade NST type) initially presented with high-grade ER-positive DCIS.

### The association between overall risk of developing a recurrent IBC event, either ipsilateral or contralateral, after primary diagnosis of DCIS and ER status

The 10-year risk of developing a recurrence episode either in the ipsilateral or contralateral breast was 17% (109/643), of which 69 events were invasive disease (11% of all cohorts and 63% of all events). Within those 69 cases, the primary DCIS was ER-positive in 55 cases (80%). Most of the recurrent events after diagnosis of ER-positive DCIS showed positive ER expression, with few events recurring as ER-negative disease (Table 4).

**Table 4:** Oestrogen receptor status of the primary DCIS and the subsequent invasive episodes, either ipsilateral or contralateral.

ER status within the primary DCIS that had subsequent invasive episode (*n* = 69, 10%)	ER status within the invasive disease (ipsilateral and/or contralateral)		
	Positive	Negative	Unknown
Positive (*n* = 55, 12%)	46 (92% of valid cases)	4 (8% of valid cases)	5
Negative (*n* = 14, 8%)	6 (43%)	8 (57%)	0

### Overall survival

The 10- and 20-year overall BC-specific death rates were 0.9% (6/643) and 1.3% (9/643), respectively. Those patients had median age of 66 years, initially presented high-grade DCIS, with comedo necrosis and half of them were treated with BCS. The ER status was positive in six cases (67%). The recurrent episode was invasive carcinoma for all cases prior to distant metastasis and death. Thus, the overall death rates after primary diagnosis of ER-positive DCIS were <0.5% and 0.7% within 10 and 20 years of the primary diagnosis of DCIS, respectively. In addition, none of the patients initially presented with low-grade DCIS, and died during the 20-year period of the study follow-up.

## Discussion

Optimal treatment of DCIS is still a controversial issue. Debate continues regarding potential overtreatment of DCIS, whether surgical excision is required for all cases and the question as to whether adjuvant therapy, RT and/or ET can be avoided for low-risk subgroups.^16^ Some multi-institutional randomised trials are underway comparing active monitoring with standard treatment for DCIS.^7,30^ The LORIS (low-risk DCIS) trial, which started in 2014, is a randomised, non-inferiority trial of comparing surgery versus active surveillance in low-risk DCIS patients. Patients with low- or intermediate-grade DCIS are randomised to either surgery or active surveillance with no hormonal treatment. The Comparison of Operative versus Medical Endocrine Therapy for Low-Risk DCIS (COMET) trial is currently ongoing and randomises low- and intermediate-grade ER-positive and HER2-negative DCIS to either standard management or active surveillance. ET use is encouraged in the active monitoring arm. The European Organisation for Research and Treatment of Cancer (EORTC)-sponsored LOw Risk DCIS (LORD) trial is due to open, and this will randomise patients with low-grade DCIS to conventional treatment versus an active monitoring strategy. The principal objective of these trials is to avoid overtreatment of low-risk DCIS, and to provide evidence that active surveillance is a management option for these patients.

In this study, there was a steady increase in the proportion of ER-positive DCIS over time, which was correlated with lower rate of HER2 positivity and higher rate of breast conservation as the primary option of DCIS management as illustrated in Fig. 1. This constellation of observed features could be a reflection of the presence of a well-established screen programme throughout the period of the study that led to increasing detection of DCIS of small size and low grade, and deceasing the rate of high-risk DCIS (based on tumour grade, size and patient age at diagnosis).^31,32^ Another possibility for higher rate of ER positivity throughout the time of the study that cannot be entirely excluded is that the tissue specimens were fresher, and the integrity of the tissue was better. However, the Nottingham cohorts of breast cancer, including DCIS, follow a standardised protocol of specimen fixation and processing in addition to tissue block storage. Based on our experience with hundreds of biomarkers tested using IHC in the invasive and the in situ diseases, no significant trend in the rate of positivity was observed between different time points, and the rate of positivity of different markers is mainly related to tumour and tissue characteristics rather than the age of the specimen or the time period of storage. Using Benchmark IHC auto-stainer and ER antibody, which is used in routine clinical workflow, to stain ER in this study reduces the possible technical errors and false staining results.

These findings were addressed in our previous work using the same cohort where we showed lower rates of high-risk DCIS over time, which was reflected by higher rate of BCS as a primary surgical management, lower rate of second operation^27^ and decreased the proportion of HER2-positive DCIS over time.^29^ Moreover, the protocol for management of DCIS in routine practice changed over time. Overall, in our series, 41% of BCS-treated patients were offered post-operative RT. However, prior to 2008, it was a common practice in our centre not to offer RT to DCIS patients with clear pathological margins 10 mm or more. Following evidence and that showed that closer margins are acceptable, the margin width was reduced, and this was followed by increasing use of RT in BCS-treated DCIS patients. Selective RT regimen was also introduced. RT was then recommended after BCS to those with high-grade DCIS, women younger than 50 years old and lesions >30 mm, regardless of tumour grade, following a multidisciplinary team discussion even if the margin is clear.^27^ In this study, there were six cases of low-grade DCIS, which showed ER expression negativity. Although this is an unusual observation, assessment of ER on TMA sections might underestimate the heterogeneity of ER expression in terms of morphological type and grade within the whole tumour. Interestingly, the PR status of these cases was negative. From our clinical experience, we came across few cases of low nuclear-grade DCIS that are ER-negative in routine practice, and the diagnosis is usually based on the cytonuclear and architecture features that were sufficient for the diagnosis of DCIS, and did not fit any other entity included in the differential diagnosis. In addition, we diagnosed occasional cases of ER-negative low nuclear-grade apocrine-type DCIS that show typical architecture pattern of DCIS. Despite the low nuclear-grade features, the cytoplasm was abundant and eosinophilic, mimicking apocrine-type cells. In our cohort, some of the low-grade DCIS that showed ER negativity had such apocrine morphology (Supplementary Fig. 2). This indicates that ER-negative low nuclear-grade DCIS exists but is extremely rare; however, the false-negative ER expression resulting from the use of TMA in this study may have exaggerated this phenomenon.

Although prognosis following a diagnosis of DCIS is excellent, the goal of ET is to reduce invasive recurrence, which occurs in up to half of the recurrent cases.^16^ Few clinical trials reported in the literature have evaluated the response of DCIS patients to ET following BCS and RT with or without commenting on ER status, either comparing tamoxifen versus placebo^20,33^ or comparing the difference of clinical benefit between tamoxifen and Anastrozole.^18,19^ For each study, updated results were published afterwards and were included in Supplementary Table 2.

In the National Surgical Adjuvant Breast and Bowel Project (NSABP) B-24 trial, all women with DCIS (*n* = 1804) received RT before being randomly assigned to ET or placebo. After a median follow-up of 6 years, a significant 37% reduction in BC recurrence was observed with ET compared with placebo. BC events were also lower in the tamoxifen-treated group (6.0%) compared with the placebo arm (9.3%) in the ipsilateral breast (*P* = 0.0009). The cumulative incidence of all IBC events in the ET group was 2.1% in the ipsilateral breast at 5 years compared with 4.2% in the placebo arm.^20^

In a retrospective evaluation of ER and PR in 732/1804 patients from the B-24 trial, 449 tumours had ER and PR measured at a central lab, while the remaining 283 tumours had results from the enrolling institutions. About 76% of DCIS was ER-positive, 24% were ER-negative. The benefit of ET by receptor status at 10 years was evaluated with an overall median follow-up of 14.5 years. Patients with ER-positive DCIS treated with ET (vs. placebo) showed significant 51% reduction in subsequent BC (ipsilateral and contralateral, invasive and non-invasive) at 10 years (HR = 0.5, *P* = 0.001). No significant benefit was observed in ER-negative DCIS.^21^ They concluded that the use of adjuvant ET (tamoxifen) offered an additional therapeutic option for patients with ER-positive DCIS.^21^

In the UK/ANZ DCIS trial, 1578 women with DCIS were randomly assigned to receive tamoxifen with or without RT. After a median of 13 years of follow-up, tamoxifen significantly reduced all new BC events by 29%, with a significant impact on ipsilateral DCIS recurrence and contralateral tumours, but no effect on ipsilateral invasive recurrence.^12,33^ ER was not an entry criterion, and there was no analysis of the invasive recurrence by ER status of the initial DCIS.

Overall, the evidence from the former studies is that ET significantly decreases BC recurrence. Based on the sub-study on B-24, this would appear to be limited to ER-positive DCIS and not ER-negative DCIS. These findings would be supported by the BC prevention studies of tamoxifen^34^ and aromatase inhibitors^35,36^ versus placebo, which have reported a significant reduction in ER-positive BC (both invasive and DCIS) but no significant reduction in ER-negative BC.

In an observational study of a prospective artificially randomised cohort,^37^ low-dose tamoxifen showed 30% reduction of any type of recurrence in women with high-risk ER-positive DCIS (*P* = 0.005). However, when ipsilateral invasive recurrence only was considered, the difference was not significant (*P* = 0.21).

However, it is noted that these studies were not large enough, nor were they designed to assess survival benefit. Importantly, the NSABP B-24 and UK/ANZ studies were on patients unselected by ER status. Although the subset of cases with known ER status in the NSABP-24 showed that adjuvant tamoxifen significantly reduced subsequent ipsilateral BC only in patients with ER-positive DCIS after standard treatment with lumpectomy and RT and not ER-negative, there was no association, in subgroup analysis, with ipsilateral invasive recurrence (*P* = 0.1) or contralateral invasive recurrence (*P* = 0.06), which are more important than the overall recurrence rate. Similar results were shown in NSABP B-35 where no obvious reduction of invasive recurrence was shown. It is noteworthy that neither of the randomised controlled trials considered DCIS grade as an inclusion criterion for patients. However, the current results showed that DCIS grade is more important than ER status to consider in prediction of recurrence risk after BCS for DCIS patients (Supplementary Fig. 3), independent of other confounding factors.

Therefore, there remains confusion regarding treatment of DCIS with one trend towards avoiding surgical treatment (and subsequently no RT) and another trend to offer more ET therapy to DCIS patients, and to make ER status assessment mandatory in all DCIS to allow clinicians to offer ET to ER-positive patients.^22,23^

Importantly, the studies that evaluated the benefit of ET therapy in DCIS considered both ipsilateral (likely a true recurrence but could be a new event) and contralateral events (a new event). Therefore, it was difficult to differentiate whether the benefits obtained by ET following the diagnosis of DCIS are related to treating the index DCIS itself to prevent its recurrence as invasive disease, or if the use of ET in these cases was more prophylactic to reduce the overall risk of invasive disease in patients with ER-positive disease. The former aims at treating DCIS as an index lesion akin to adjuvant ET of invasive disease, whereas the latter aims at treating DCIS as a marker of subsequent risk.^38^ The results of the current study support the fact that ER status of the primary DCIS correlates strongly with the ER status of recurrent invasive disease in the same or contralateral breast, and therefore justifies the use of ET in these patients. On the other hand, ER-negative DCIS is associated with development of ER-negative invasive disease, so ET is unlikely to influence the risk of invasive disease development. We also showed that RT in general, and RT regardless of the ER status, offered to ER-positive DCIS cases has improved outcome after BCS. Therefore, the current results support NICE guidelines to provide ET for patients with high-risk ER-positive DCIS when RT cannot be given for any reason.^22^ In ER-negative DCIS, RT reduces the risk of recurrence from 28% (9/32) in patients who did not receive RT, to 19% (5/26) in patients who receive post-operative RT; however, this difference was not statistically significant likely due to the small number of ER-negative cases included in the analysis.

In this study, our results showed that there is no significant association between ER status and the development of an ipsilateral breast event, either as DCIS or invasive recurrence. This was similar when we carried out the analysis on BCS-treated patients only, or when we included the whole cohort, regardless of surgical management. We focused mainly on BCS group as they are the group of interest in NICE guidelines. The rate of recurrence after mastectomy was very low (1.2% recurrence rate that was ~6% of all recurrences in the study cohort). Patients treated with mastectomy usually receive no further therapy (neither RT nor ET); thus, they are not included under the recommendation of NICE guidelines. The current finding is similar to that of the sub-study of NSABP B-24^21^ and also of other studies,^37,39^ where the recurrence rates in the placebo group were similar for both ER-positive and ER-negative DCIS. We also showed that ER was not a contributing factor affecting the development of CBC in DCIS patients.

It is important to rationalise the use of prophylactic or preventive therapy considering the side effects,^40,41^ cost and the magnitude of risk when planning such therapies; patients who are at high risk of disease development or those likely to develop high-risk disease will derive the most benefit from such therapy. The results in this study showed that low-grade DCIS treated with BCS, had excellent prognosis with 100% survival rate even in occasional patients who developed invasive disease, which was low-grade ER-positive invasive carcinoma. In addition, all ER-positive DCIS that recurred as invasive disease and showed subsequent BC-related mortality incidence were of high grade, and they were treated with BCS only. Therefore, we consider that adjuvant ET for low-risk DCIS is questionable as in view of the excellent outcome event in the recurrent cases. In RTOG 9804, there are so few events at the time of analysis that the data could not support or refute the role of ET in the treatment of low-risk DCIS.^42^ Another important finding in this study is the difference between low–nuclear-grade DCIS and the intermediate- and high–nuclear-grade DCIS group of patients regarding the development of invasive carcinoma risk, and that the binary distinction of DCIS for risk stratification should be between low- and intermediate-/high–nuclear-grade group rather than between low-/intermediate- and high–nuclear-grade groups of DCIS.

This study has some potential limitations. None of the patients included in this cohort were offered adjuvant ET. Management of DCIS was following local institutional protocols, and this was in line with the local and national UK guidelines present at the time of the study. The National Comprehensive Cancer Network (NCCN) and NICE guidelines for offering ET in DCIS were published in 2017 and 2019, respectively,^22,43^ which were not available during the period of the study. Second, the study was conducted on DCIS cases spanning a long time period with potential bias regarding the availability of tumour tissue for ER assessment. Thirdly, ER was evaluated on TMA sections that might underestimate the heterogeneity of ER expression within the whole tumour. The interobserver variability in DCIS grading is another potential limitation. Although DCIS grading similar to other biological features of differentiation is subjective, and distinction between intermediate- and high-grade DCIS is often challenging, diagnosis of low-grade DCIS is considered to be the easiest and more objective.

## Conclusions

There is a tendency for overtreatment of DCIS, which can be by surgery and/or RT and/or ET. Although the impact of ET on the overall survival of DCIS patients is not demonstrated, ET reduces the risk of development of an invasive disease by up to 40%. Recommending ET to DCIS patients who do not receive RT can be justifiable; however, this should be based on ER status (positive) and grade (intermediate and high) of DCIS. ET appears to reduce the risk of invasive disease in general, and not just limited to reducing the incidence of recurrence of the index DCIS, and as such, its use may contrast with the purpose of its use in IBC patients. The routine management of all DCIS, including the low-risk group with ET, does not appear to be justified by current data. There is an urgent need for molecular biomarkers and evidence-based guidelines to further refine the recurrence risk assessment and treatment decision-making in DCIS patients.

## Supplementary information

Supplementary Tables and Figures

## Data Availability

The authors confirm that data that have been used are available on reasonable request.
