# Correction: The clinical significance of oestrogen receptor expression in breast ductal carcinoma in situ

**DOI:** 10.1038/s41416-020-01097-9

**Published:** 2020-09-16

**Authors:** Islam M. Miligy, Michael S. Toss, Sho Shiino, Georgette Oni, Binafsha M. Syed, Hazem Khout, Qing Ting Tan, Andrew R. Green, R. Douglas Macmillan, John F. R. Robertson, Emad A. Rakha

**Affiliations:** 1 Division of Cancer and Stem Cells, School of Medicine, University of Nottingham Biodiscovery Institute, The University of Nottingham, Nottingham, UK; 2 The Breast Institute, Nottingham City Hospital, Nottingham University Hospitals NHS Trust, Nottingham, UK; 3 Medical Research Centre, Liaquat University of Medical & Health Sciences, Jamshoro, Pakistan; 4 Division of Graduate Entry Medicine, School of Medicine, University of Nottingham Royal Derby Hospital, Derby, UK

**Keywords:** Breast cancer, Breast cancer

**Correction to**: *British Journal of Cancer* (2020) 10.1038/s41416-020-1023-3, published online 10 August 2020

Since the publication of this paper the authors noticed an error in the name of Dr Qing Ting Tan, which was incorrectly listed as Quing Tan. This has been corrected above.

