# Supplementary material for: The clinical significance of oestrogen receptor expression in breast ductal carcinoma in situ
Source: Br J Cancer. 2020 Aug 10;123(10):1513–20. doi: 10.1038/s41416-020-1023-3 (PMC7653904; doi:10.1038/s41416-020-1023-3)
Supplement: Supplementary file 1 — Supplementary Tables and Figures [file 41416_2020_1023_MOESM1_ESM.docx]

**Supplementary Tables**

**Supplementary Table 1:** Multivariate analysis of various variables associated with DCIS recurrence in patients treated with breast conserving surgery

|  | Hazard Ratio | 95.0% CI for Exp(B) | | P value |
| --- | --- | --- | --- | --- |
|  |  | Lower | Upper |  |
| Patient age at diagnosis | 1.5 | 1.3 | 1.8 | **0.008** |
| DCIS size | 1.5 | 0.8 | 2.5 | 0.170 |
| DCIS Grade | 1.5 | 1.1 | 2.2 | **0.027** |
| Radiotherapy | 0.4 | 0.2 | 0.9 | **0.018** |
| Oestrogen receptor status | 0.9 | 0.5 | 1.9 | 0.985 |

Significant p values are in **bold**

**Supplementary Table 2:** Clinical trials for the role of hormonal therapy in DCIS after breast conserving treatment.

| **Study** | **ER status as inclusion criterion** | **Primary End points** | **Outcome of the study** |
| --- | --- | --- | --- |
| Radiotherapy (RT) and Tamoxifen in women with completely excised ductal carcinoma *in situ* of the breast in the UK, Australia, and New Zealand: randomised controlled trial [33]*. | No prior knowledge of the hormone receptor status of the patients. | To compare the efficacy of complete local excision alone with excision followed by RT to the residual ipsilateral breast, or excision followed by Tamoxifen for 5 years, or both, in reducing the incidence of subsequent ipsilateral invasive breast carcinoma in patients with DCIS. | Ipsilateral invasive disease was not reduced by Tamoxifen, but recurrence of overall DCIS was decreased. RT reduced the incidence of ipsilateral invasive disease and ipsilateral DCIS, but there was no effect on the occurrence of contralateral disease. There was no evidence of interaction between RT and Tamoxifen. |
| Tamoxifen in treatment of intraductal BC: National Surgical Adjuvant Breast and Bowel Project B-24 randomised controlled trial [20]*. | No prior knowledge of the hormone receptor status of the patients. | Occurrence of invasive or non-invasive tumours in the ipsilateral or contralateral breast following Tamoxifen therapy. | The effectiveness of Tamoxifen in treating DCIS without RT is speculative.  The value of Tamoxifen used in combination with RT to lower the occurrence of invasive cancer could justify the suggestion that combined therapy replaces mastectomy for the treatment of DCIS patients in whom radiological findings are unlikely to be related to an invasive tumour |
| Adjuvant Tamoxifen reduces subsequent BC in women with oestrogen receptor-positive ductal carcinoma in situ: a study based on NSABP protocol B-24 [21]*. | ER and PR evaluation were carried out later for a subset of cases. | Time to the occurrence of any BC as a first event ipsilateral or contralateral subsequent to the original diagnosis of DCIS. | Adjuvant Tamoxifen significantly reduced subsequent ipsilateral BC only in patients with ER-positive DCIS after standard treatment with lumpectomy and RT. Tamoxifen reduced contralateral BC in patients with ER-positive and -negative DCIS. No ipsilateral benefit was observed in ER-negative disease. |
| Primary results, NSABP B-35/NRG Oncology: A clinical trial of Anastrozole vs Tamoxifen in postmenopausal patients with DCIS  undergoing lumpectomy plus RT A randomized clinical trial  [18]*. | ER or PR positive | BC-free interval, the time from randomisation to any BC event including local, regional, or distant recurrence or contralateral disease, invasive or DCIS. | There was no significant decrease in ipsilateral cancer, either invasive or non-invasive. |
| Anastrozole versus Tamoxifen for the prevention of locoregional and contralateral BC in postmenopausal women with locally excised ductal carcinoma in situ (IBIS-II DCIS): a double-blind, randomised controlled trial [19]*. | ER and PR positivity were determined as greater than or equal to 5% positive cells (equivalent of Quick- score of three or above and H-score of ten or above). | The development of histologically confirmed BC, both invasive and new or recurrent DCIS. | No clear efficacy differences were seen between the two treatments. |

BC=Breast cancer, RT=Radiotherapy

*Reference numbers are listed as per their order in the main manuscript.

**Supplementary Figures**


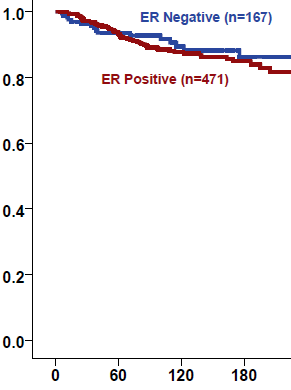


**p value at 120 months =0.721**

**p value at 180 months = 0.540**

**Patients at risk**

**471 365 220 93**

**167 117 76 39**

**Supplementary Figure 1:** Kaplan-Meier curve shows the association between ER expression and ipsilateral local recurrence rate in the whole cohort regardless the surgical treatment (Horizontal axis: local recurrence free interval in months, vertical axis: probability of recurrence).


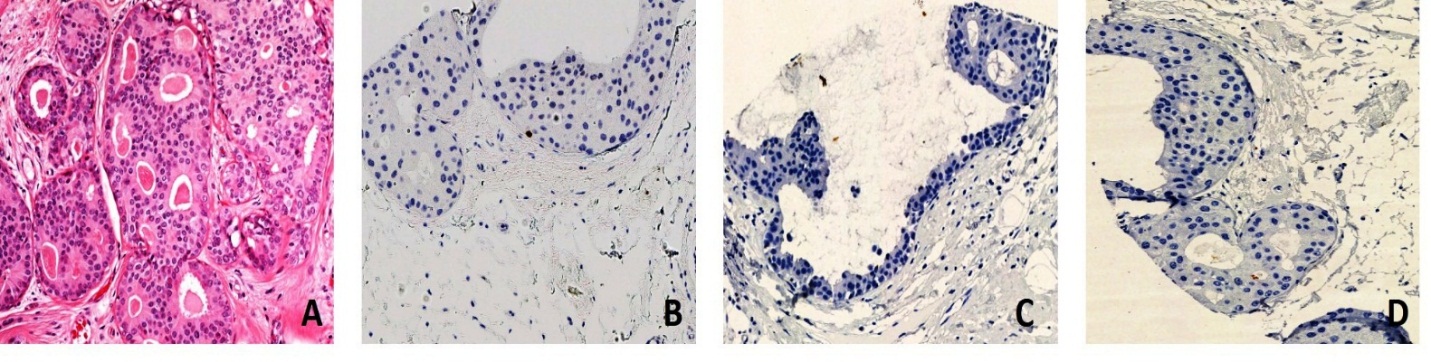


**Supplementary Figure 2:** Photomicrographic examples of low-grade DCIS that show negative expression of oestrogen receptor (ER); A) H&E stained section, B-D) immunohistochemistry stained sections showing negative ER expression. Note the apocrine changes (abundant esinophilic cytoplasm) and low-grade morphology.

| **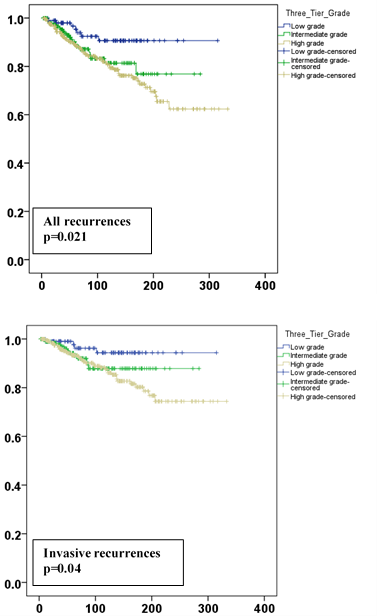** |
| --- |

**Supplementary Figure 3:** Kaplan-Meier curve shows the association between DCIS grade and ipsilateral local recurrence (all recurrence in the upper curve and invasive recurrence in the lower curve). (Horizontal axis: local recurrence free interval in months, vertical axis: probability of recurrence).
